# Case Report: Hemorrhagic fever with renal syndrome caused by Seoul orthohantavirus mimicking severe acute pancreatitis: dynamic imaging evolution and pathogen confirmation by mNGS

**DOI:** 10.3389/fmed.2026.1995402

**Published:** 2026-10-28

**Authors:** Xuanqi Liu, Liting Song

**Affiliations:** 1 Intensive Care Department, The Affiliated Traditional Chinese Medicine Hospital, Guangzhou Medical University, Guangzhou, China; 2 General Surgery, The Affiliated Traditional Chinese Medicine Hospital, Guangzhou Medical University, Guangzhou, China

**Keywords:** acute pancreatitis, case report, hemorrhagic fever with renal syndrome, metagenomic next-generation sequencing, Seoul orthohantavirus

## Abstract

**Background:**

Hemorrhagic fever with renal syndrome (HFRS) is a zoonotic disease caused by orthohantaviruses, characterized by fever, thrombocytopenia, renal impairment, and capillary leak syndrome. Its atypical presentations can closely mimic acute pancreatitis (AP), posing substantial diagnostic challenges.

**Case presentation:**

A 33-year-old man presented with fever, vomiting, and watery diarrhea. Initial computed tomography (CT) revealed peripancreatic and perirenal exudation suggestive of severe acute pancreatitis. He already had profound thrombocytopenia on admission (nadir 29  ×  10⁹/L), progressive renal impairment, hematuria, and proteinuria, without typical pancreatitis risk factors or elevated pancreatic enzymes. Contrast-enhanced CT demonstrated progressive retroperitoneal exudation with relatively mild pancreatic parenchymal changes and newly developed bilateral pleural effusion. Whole-blood metagenomic next-generation sequencing (mNGS) detected high-copy Seoul orthohantavirus, with concurrent hepatitis B virus. The patient recovered fully with supportive care, intravenous immunoglobulin, and symptomatic treatment; pre-discharge ultrasound showed normal pancreatic morphology.

**Conclusion:**

Seoul orthohantavirus infection can closely mimic severe acute pancreatitis clinically and radiologically. Disproportionate thrombocytopenia, renal involvement, and progressive retroperitoneal exudation with preserved pancreatic parenchyma are key diagnostic clues, and mNGS enables early definitive pathogen confirmation, facilitating appropriate management.

## Introduction

### Case presentation and clinical data

A 33-year-old man presented with one week of recurrent fever, vomiting, and watery diarrhea. He had no history of gallstones, hypertriglyceridemia, alcohol abuse, or other recognized risk factors for acute pancreatitis. Although viral gastroenteritis could explain fever, vomiting and watery diarrhea, the presence of epigastric tenderness on physical examination together with marked peripancreatic-perirenal exudates identified on initial abdominal CT raised strong clinical suspicion for severe acute pancreatitis. Initial non-contrast abdominal CT showed peripancreatic and perirenal haziness, renal fascial thickening, and small-volume abdominopelvic effusion with mild pancreatic swelling, findings highly suggestive of severe acute pancreatitis (Figure 1).

**Figure 1:**
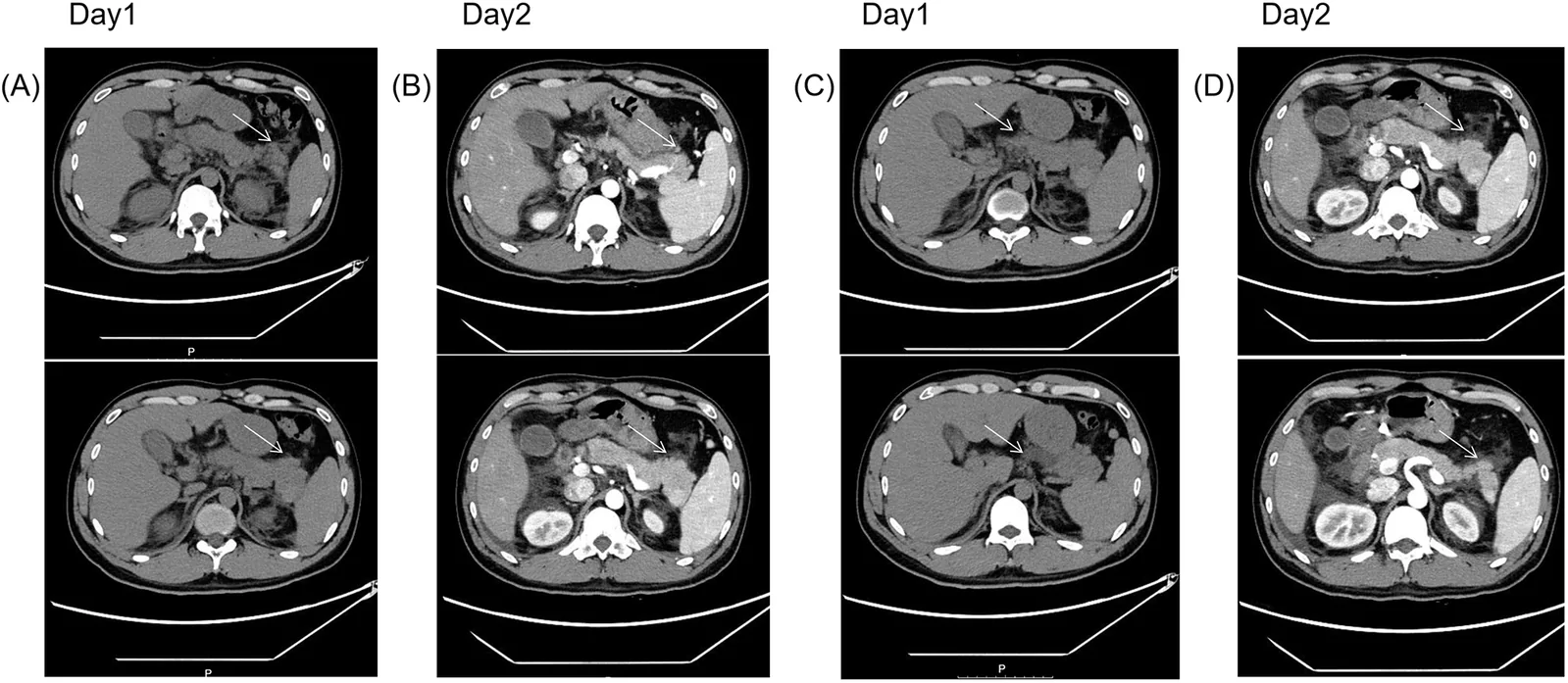
Serial axial abdominal computed tomography (CT) images of Seoul orthohantavirus-associated capillary-leak syndrome mimicking severe acute pancreatitis. **(A, C)** Non-contrast abdominal CT acquired on hospital day 1 (admission). White arrows indicate the pancreas, peripancreatic fat-stranding inflammatory exudates, and thickened bilateral prerenal fascia with perirenal exudates. These radiological features initially raised high clinical suspicion for severe acute pancreatitis. **(B, D)** Contrast-enhanced abdominal CT acquired on hospital day 2. Compared with hospital day 1 non-contrast CT, retroperitoneal, peripancreatic and perirenal exudates are obviously progressive. After contrast administration, the pancreatic parenchyma shows no definite necrosis or large-scale parenchymal destruction.

Initial supportive measures for suspected acute pancreatitis were implemented, including intravenous proton-pump inhibitor, octreotide infusion, empirical broad-spectrum antibiotics, and volume resuscitation. Of note, severe thrombocytopenia (nadir 29 × 10⁹/L) was already documented on admission rather than developing during hospitalization. However, the patient demonstrated progressive renal impairment, hematuria, and proteinuria, without biochemical elevation of pancreatic enzymes.

Two abdominal CT examinations were performed: a non-contrast CT on hospital day 1 (admission), followed by contrast-enhanced abdominal CT on hospital day 2. Contrast-enhanced CT revealed progressive retroperitoneal exudation accompanied by newly developed bilateral pleural effusion and splenomegaly.

Routine work-up for undifferentiated fever was sent before mNGS testing, including bacterial blood cultures, stool culture, and a respiratory-borne viral screening panel, all of which returned negative results. Thrombotic microangiopathy (TMA) laboratory screening was not performed because clinical suspicion for TMA was low: the patient lacked schistocytes on peripheral blood smear, had no severe neurologic manifestations, and mNGS rapidly identified Seoul orthohantavirus as the causative pathogen.

Whole-blood mNGS detected high-copy Seoul orthohantavirus, with concurrent hepatitis B virus (HBV) and no bacterial or fungal pathogens. Hepatoprotective therapy consisted of intravenous reduced glutathione and magnesium isoglycyrrhizinate. Due to persistent vomiting, abdominal distension, and unstable clinical status precluding oral feeding, the patient was kept nil per os (NPO) at admission, not as a standard mandatory intervention for suspected acute pancreatitis. Enteral nutrition was gradually initiated once abdominal symptoms improved. After supportive care, intravenous immunoglobulin, albumin supplementation, hepatoprotective therapy, and electrolyte correction, the patient recovered fully; pre-discharge ultrasound showed normal pancreatic morphology (Figure 2).

**Figure 2:**
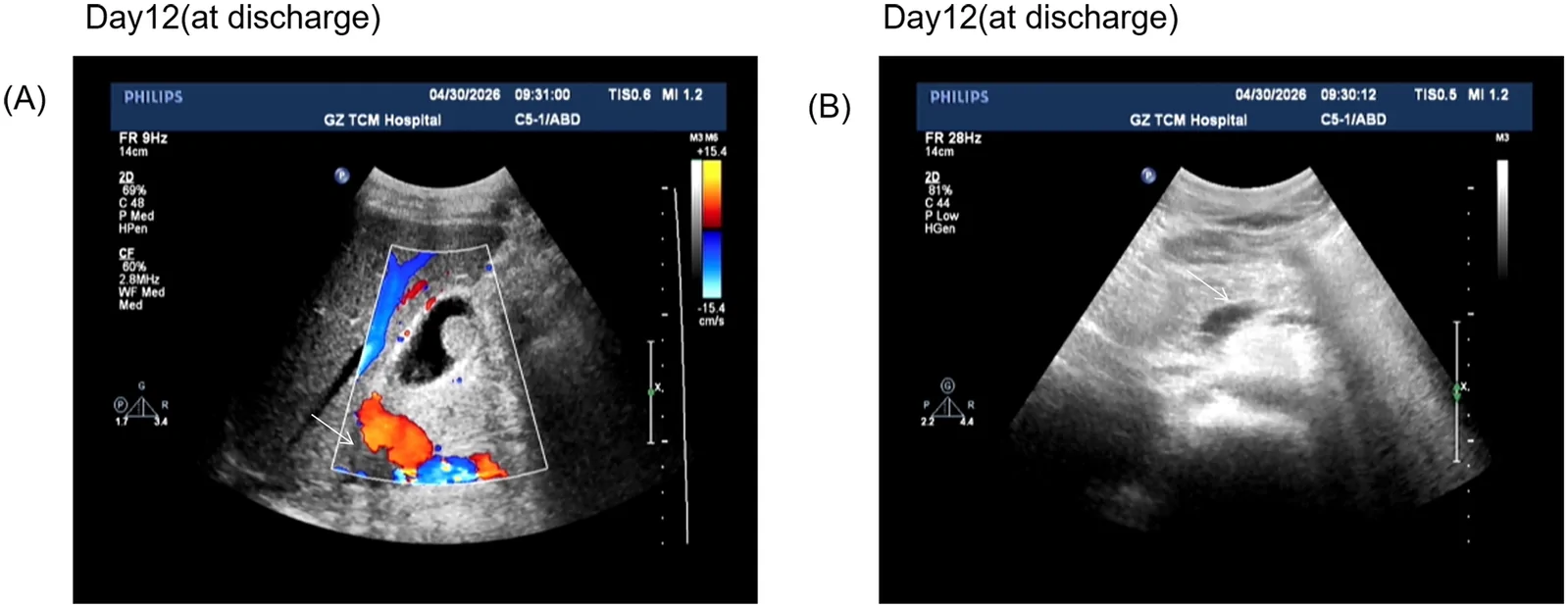
Pre-discharge abdominal ultrasound obtained on hospital day 12 (at discharge). **(A)** Color-Doppler ultrasound view of upper abdomen. **(B)** Grayscale two-dimensional ultrasound image. White arrows mark the pancreatic gland. The pancreas presents normal parenchymal echotexture, without swelling, enlargement or surrounding fluid-collection, confirming complete resolution of previous retro-peritoneal inflammatory exudative lesions.

### Laboratory and imaging findings

Initial laboratory investigations showed a mixed acid-base disturbance, characterized by respiratory alkalosis with normal anion-gap (AnGap 2-10 mmol/L),hyponatremia, hypokalemia, and liver function injury. The most striking abnormality was profound thrombocytopenia (29 × 10⁹/L); urinalysis revealed hematuria and proteinuria.

Table 2 was revised to add serum amylase, lipase, serum triglyceride, serum urea, serum creatinine, sodium, potassium, hemoglobin values. Reticulocyte count was not tested in clinical practice; hemoglobin levels over serial time-points are presented to evaluate for bone-marrow suppression. Urine sediment examination did not demonstrate granular or hyaline casts. Serial urine output was prospectively recorded as part of intensive care monitoring; overall urine output remained adequate throughout hospitalization without anuria or oliguric renal failure. Mild anemia was observed (minimum hemoglobin 127 g/L).

Serial changes in leukocytes, high-sensitivity C-reactive protein, neutrophil and lymphocyte percentages, transaminases, platelets, albumin, and coagulation parameters are summarized in Table 2. The dynamic clinical, laboratory, imaging, and management course across hospitalization is summarized in the diagnostic timeline (Table 1).

**Table 1:** Diagnostic timeline.

Time point	Clinical manifestations	Key laboratory findings	Imaging & pathogen test	Clinical management & decision-making
One week before admission	Low-grade intermittent fever, lumbago; symptom recurred after outpatient infusion	Not available	No imaging performed	Symptomatic infusion at local clinic
One day before admission	High fever (peak 39.3 °C), anorexia, vomiting, watery diarrhea >10 times/day	Not available	Not done	Emergency department visit; patient initially refused hospitalization
Hospital day 1 (admission)	Alert, fatigued, epigastric mild tenderness, no rebound tenderness; vomiting and watery diarrhea	PLT 29 × 10⁹/L (critical); mixed acid-base disorder,(respiratory alkalosis, normal anion-gap), hyponatremia, hypokalemia; liver function impairment; urine: hematuria 2+, proteinuria 1+,urine output adequate	Non-contrast abdominal CT: peripancreatic/perirenal exudates, renal fascia thickening, abdominopelvic small-volume effusion; mild pancreatic swelling	Suspected severe acute pancreatitis; NPO due to vomiting and abdominal distension, octreotide infusion, PPI, empirical antibiotics, fluid resuscitation; critical thrombocytopenia reported
Hospital day 2	Persistent abdominal distension and pain, low-grade fever	PLT remained low; elevated liver enzymes, rising serum creatinine	Contrast-enhanced CT: progressive retroperitoneal exudates; new-onset bilateral small pleural effusion; splenomegaly	Transferred to intensive monitoring; whole-blood sample sent for mNGS; continued supportive treatment
Hospital day 3	Abdominal pain partially relieved; no fever	PLT beginning to recover; ALT, AST, Cr still elevated; hypoalbuminemia	mNGS result: high-copy Seoul orthohantavirus, concomitant HBV; bacteria/fungi negative	HFRS confirmed; intravenous immunoglobulin, human albumin, hepatoprotective therapy, electrolyte correction
Hospital days 6–9	No abdominal pain/vomiting; bowel movements normal; afebrile	Gradual improvement of PLT, liver enzymes, creatinine, inflammatory markers	No repeated CT	Tapering supportive treatment; switched to oral medication; gradual dietary advancement to full-liquid diet
Hospital day 12 (discharge)	Asymptomatic; good appetite, normal defecation	Liver enzymes mildly elevated; other main indices near reference range	Abdominal ultrasound: normal pancreatic morphology; mild fatty liver; no pancreatic swelling or peripancreatic fluid	Discharged; outpatient follow-up scheduled for liver function and HBV monitoring

### Hospital course and management

The initial working diagnosis was suspected severe acute pancreatitis, and management comprised nil-per-os status due to patient intolerance of oral intake, octreotide infusion, a proton-pump inhibitor, empirical antimicrobial therapy, and fluid resuscitation. When the severe thrombocytopenia and renal dysfunction could not be explained by primary pancreatitis, HFRS was considered. On hospital day 3, whole-blood mNGS returned high-copy Seoul orthohantavirus, confirming the diagnosis. Treatment was adjusted to include intravenous immunoglobulin, human albumin, hepatoprotective therapy, and electrolyte correction. Abdominal pain resolved, and platelet count, liver, and renal parameters gradually normalized. The patient was discharged on hospital day 11 in good clinical condition, with outpatient follow-up scheduled for monitoring of liver function and HBV.

## Discussion

This case highlights a diagnostically challenging presentation in which Seoul orthohantavirus infection clinically and radiologically mimicked severe acute pancreatitis, underscoring the need to consider HFRS in the differential diagnosis of unexplained pancreatitis-like presentations (1). Acute pancreatitis is a recognized but rare complication of HFRS (2, 6), and its distinction from hantavirus-induced pancreatic involvement is notoriously difficult because of overlapping clinical and imaging features (9). The present case further illustrates that peripancreatic exudation and retroperitoneal fluid—hallmark findings of severe acute pancreatitis—can also result from the profound systemic capillary leakage and retroperitoneal edema characteristic of hantavirus infection (4, 5). In this context, marked thrombocytopenia, renal impairment with hematuria and proteinuria, and the absence of typical pancreatitis risk factors constituted pivotal clues that prompted consideration of an alternative etiology (9). Moreover, the normal pancreatic enzyme levels observed here demonstrate that biochemical markers alone cannot reliably exclude HFRS in suspected pancreatitis (10).

It should be acknowledged that severe acute pancreatitis itself may produce thrombocytopenia and renal dysfunction secondary to systemic inflammation. However, several features argued against primary acute pancreatitis in this patient: thrombocytopenia was already present at initial presentation as a critical-value finding before any expected inflammatory peak of pancreatitis; renal injury was accompanied by prominent urinary findings (hematuria and proteinuria) typical for hantavirus-mediated tubular injury rather than purely pre-renal renal impairment secondary to pancreatitis-related hypovolemia; and serum amylase/lipase remained within normal reference ranges throughout admission despite extensive peripancreatic exudates on CT imaging.

The dynamic imaging feature observed in this case—progressive retroperitoneal exudation on contrast-enhanced CT accompanied by relatively mild pancreatic parenchymal changes—merits particular attention in the differential diagnosis of hantavirus infection presenting with abdominal manifestations. While AP is a recognized complication of HFRS, the typical CT findings in such cases commonly include pancreatic enlargement, peripancreatic edema, and occasionally necrosis or hemorrhage (4). However, extensive retroperitoneal involvement with a relatively preserved pancreatic gland reflects the underlying pathophysiological hallmark of hantavirus infection—virus-induced increased microvascular permeability leading to fluid extravasation into the retroperitoneal space. Indeed, the retroperitoneum has been identified as a major site of vascular leak in HFRS (11), and established evidence indicates that hantavirus elicits a robust inflammatory cascade with cytokine release and endothelial dysfunction that can produce extensive retroperitoneal fluid accumulation even in the absence of true pancreatic necrosis (12, 13). Prior case reports of HFRS mimicking acute pancreatitis have generally relied on single-time-point scans and could not capture this evolving radiological signature (7). Recognition of this distinctive pattern is of substantial practical importance, particularly in endemic regions (14, 15). From a clinical standpoint, progressive retroperitoneal exudation with relatively mild pancreatic changes on follow-up CT, when accompanied by thrombocytopenia and renal dysfunction, should prompt clinicians to broaden the differential beyond pancreatic pathology.

Attributing the initial retroperitoneal exudation and abdominopelvic effusion to severe acute pancreatitis follows a conventional diagnostic pathway, given that such radiological findings are well-recognized features of this condition. However, the subsequent constellation of profound thrombocytopenia, progressive renal impairment, and urinary abnormalities extended beyond the typical scope of pancreatic pathology and prompted re-evaluation of the initial diagnostic assumption (9). This scenario illustrates a classic diagnostic pitfall, where an initially plausible diagnosis supported by compelling imaging can lead to anchoring bias that delays consideration of alternative etiologies with similar radiological features. Indeed, various infections mimic the clinical and radiological presentation of acute pancreatitis, including viral pathogens that also cause systemic thrombocytopenia and renal dysfunction (16, 17); beyond pancreatitis, hantavirus infection can mimic other acute abdominal emergencies such as acute appendicitis (18, 19). The absence of typical inciting factors—such as gallstones(serum triglyceride level serial data now available in Table 2, maximal triglyceride 3.90 mmol/L, not fulfilling criteria for hypertriglyceridemic-induced pancreatitis)or hypertriglyceridemia—and the lack of significant pancreatic enzyme elevation further challenged the primary diagnosis (9).

**Table 2:** Dynamic changes of main laboratory indicators and corresponding normal reference ranges during hospitalization (*revised table: newly-added items: amylase, lipase, triglyceride, urea, creatinine, sodium, potassium, hemoglobin).

Laboratory Parameter	Normal Reference Range	Day1 (Admission)	Day2	Day4	Day6	Day9	Day12
Leucocytes (10⁹/L)	4.0–10.0	13.29	12.88	13.76	10.57	8.18	–
hsCRP (mg/L)	0.0–1.0	–	19.1	23.3	20.2	6.0	2.1
Neutrophils (%)	50.0–70.0	49.4	38.4	55.4	60.7	59.8	–
Lymphocyte (%)	20.0–40.0	39.0	46.9	28.8	27.4	27.2	–
Monocyte percentage (%)	3.0–10.0	11.4	14.3	15.0	10.4	11.2	10.7
ALT (U/L)	5–40	187.0	197.0	133.0	81.0	71.0	59.0
AST (U/L)	8–40	144.0	–	95.0	92.0	100.0	79.0
Platelets (10*9/L)	125–350	29.0	100.0	166.0	312.0	351.0	358.0
ALB (g/L)	35.0–50.0	26.5	25.2	29.0	31.6	39.1	–
BNP (pg/mL)	＜125.0	115.0	–	–	–	–	–
APPT(s)	25.1–36.5	48.3	36.8	–	–	–	–
Serum amylase (U/L)	35–135	78	–	82	–	–	–
Serum lipase (U/L)	13–60	44	–	49	–	–	–
Triglyceride (mmol/L)	0.45–1.69	1.81	–	3.90	2.78	2.76	–
Urea (mmol/L)	3.10–8.00	4.30	–	8.44	5.21	4.56	–
Creatinine (*μ*mol/L)	57.0–97.0	71.0	–	146.0	131.0	113.0	–
Sodium (mmol/L)	137–147	126.1	124.6	142.4	143.5	145.1	–
Potassium (mmol/L)	3.5–5.5	3.21	3.10	3.42	3.40	3.70	–
Hemoglobin (g/L)	130–175	156.0	139.0	127.0	127.0	137.0	135.0
Temperature (℃)	36.0–37.2	39.3	38.4	37.7	37.2	37.0	36.8

hsCRP, high-sensitivity C-reactive protein; ALT, alanine aminotransferase; AST, aspartate aminotransferase; ALB, albumin; APTT, activated partial thromboplastin time; “–” indicates no test on that day.

Additional note: Reticulocyte count was not obtained clinically; hemoglobin serial values are presented to evaluate for severe bone-marrow suppression. Urine sediment analysis: hematuria 2+, proteinuria1+, without granular or hyaline casts. Serial urine output was adequate, no oliguria/anuria occurred. Mixed acid-base disturbance was respiratory alkalosis with normal anion-gap.

Bone-marrow suppression could not be fully excluded without reticulocyte testing; nevertheless, serial hemoglobin values did not show progressive severe anemia, which makes profound isolated bone-marrow suppression less likely in this clinical picture.

The clinical profile of this patient—marked fever preceding gastntestinal symptoms, severe thrombocytopenia disproportionate to pancreatic injury, renal dysfunction with hematuria and proteinuria, and the absence of classic pancreatitis risk factors—aligns closely with previously documented patterns of hantavirus infection masquerading as pancreatic pathology. Persistent fever has consistently been identified as an early prodromal feature that predates abdominal complaints in patients with HFRS (20, 21), a sequence mirroring the chronological symptom evolution observed here. Likewise, disproportionately low platelet counts without evidence of bone marrow suppression or disseminated intravascular coagulation have been emphasized as a key hematologic clue distinguishing HFRS from primary pancreatic or biliary etiologies (10, 20). Renal involvement manifesting as proteinuria and hematuria is a hallmark of orthohantavirus infection even in atypical presentations (10). The absence of gallstones, hypertriglyceridemia, or alcohol consumption—factors accounting for most acute pancreatitis cases—has likewise been underscored in previous reports of HFRS mimicking pancreatitis (4). Radiologically, extensive retroperitoneal or peripancreatic exudation with relatively preserved pancreatic parenchyma has been documented in hantavirus-infected patients (5). Furthermore, normal or only mildly elevated pancreatic enzyme levels despite severe abdominal symptoms have been repeatedly observed in such patients, reinforcing the diagnostic challenge posed by this overlap syndrome (10).

The concurrent detection of hepatitis B virus (HBV) raises the question of its potential contribution to the pancreatic injury. Viral infections are a rare but established cause of acute pancreatitis (3, 22), with a wide spectrum of pathogens implicated, including mumps virus, coxsackievirus, cytomegalovirus, and hepatitis viruses (22). Specifically, case reports and series have documented an association between hepatitis B and pancreatitis, although the pathogenic mechanism remains incompletely understood (23, 24). In a prospective study of 124 patients with acute viral hepatitis and severe abdominal pain, 7 (5.65%) were diagnosed with acute pancreatitis, one attributed to hepatitis B (24); another study from Nepal reported acute pancreatitis in 25 of 382 patients with acute viral hepatitis, though those cases were exclusively due to hepatitis E and A viruses (25). More broadly, viral infection ranks among the leading etiologies of acute pancreatitis in children (26). However, the present case diverges substantially from the typical profile of HBV-associated pancreatitis: the patient had only mild liver enzyme elevation without jaundice or other signs of acute viral hepatitis, whereas most reported HBV-related cases occurred in the setting of clinically overt acute hepatitis (24). Moreover, a study of 66 patients with acute Puumala hantavirus infection found no cases of acute pancreatitis despite frequent liver enzyme abnormalities (27). The high-copy Seoul orthohantavirus detected by mNGS, together with the characteristic sequence of fever, thrombocytopenia, renal impairment, and capillary leakage, provides a cohesive pathophysiological framework that accounts for the entire clinical course. The co-detected HBV, while a potential contributory factor, does not sufficiently explain the hematological and renal abnormalities that were the hallmark features of this case. Thus, HBV co-infection may have played at most an adjunctive role, and Seoul orthohantavirus is the principal etiological agent.

Notably, inverse temporal trends between C-reactive protein and serum albumin were observed during hospitalization. Elevated CRP reflected acute-phase inflammatory response triggered by hantavirus infection, while hypoalbuminemia was multifactorial, secondary to systemic capillary leak syndrome, reduced hepatic synthesis, and protein extravasation into retroperitoneal and serous body cavities. Albumin gradually increased as capillary leakage resolved and inflammatory markers declined during clinical recovery.

Regarding renal pathophysiology in HFRS: Orthohantavirus infects renal tubular endothelial cells, triggering local inflammation, increased vascular permeability, interstitial edema, and tubulo-interstitial nephritis. In our patient, urinalysis showed proteinuria and hematuria; urine sediment was negative for granular or hyaline casts. Clinical course did not develop severe renal tubular acidosis. Renal impairment was reversible, consistent with typical HFRS-associated tubulo-interstitial injury.

Although mNGS offers a highly sensitive approach to pathogen detection, its output is inherently shaped by the bioinformatic pipelines and reference databases employed during analysis, which can influence the final microbial interpretation (8, 28). Its performance is not uniformly superior: targeted multiplex amplification methods have shown better overall sensitivity than mNGS for detecting respiratory viruses (29), and mNGS sensitivity can be lower for specific organisms, such as a combined sensitivity of 0.69 for Mycobacterium tuberculosis complex detection in a meta-analysis (30) and reduced diagnostic accuracy for fungal infections in immunocompromised patients with suspected pneumonia (31). Moreover, the high sensitivity of mNGS may detect multiple organisms simultaneously, complicating the differentiation between true pathogens and innocent bystanders or contaminants (29). Therefore, although mNGS provides an unbiased and rapid survey of potential pathogens, its results should be interpreted alongside serological assays and conventional microbiological tests to establish a definitive diagnosis and delineate the contribution of co-infections (32).

Despite its clinical value, this report has several limitations that merit consideration. The diagnosis was singularly dependent on whole-blood mNGS for pathogen confirmation; serological assays—the cornerstone of conventional hantavirus diagnostics—were not performed, precluding comprehensive immunological corroboration. Furthermore, this is a solitary case report, so the generalizability of the observed clinico-radiological patterns to the broader population of HFRS patients presenting with abdominal manifestations is inherently constrained, and the dynamic radiological evolution was derived from a single patient's imaging series. In addition, although mNGS identified concurrent HBV, its potential contribution to the hepatic dysfunction or overall clinical course could not be definitively delineated, adding confounding complexity. Additional limitation: Reticulocyte testing and full TMA panel were not performed due to real-world clinical practice constraints. These limitations notwithstanding, the present case highlights diagnostic challenges and underscores the need for future multicenter studies to systematically characterize the clinical phenotype and optimal diagnostic algorithm for HFRS mimicking a surgical abdomen, incorporating standardized serological and molecular testing across a larger patient sample (33).

The clinical mimicry observed in this case underscores a diagnostic challenge that extends beyond individual patient management. Hantavirus infections frequently present with gastrointestinal symptoms and abdominal pain that can obscure the classic renal and hemorrhagic manifestations, leading to initial misdiagnosis, particularly in non-endemic regions where clinical suspicion may be low despite suggestive laboratory abnormalities such as thrombocytopenia and proteinuria (15). Given that AP is a recognized and severe complication of HFRS with a poor prognosis (4), and that serial monitoring often reveals a characteristic pattern of resolving thrombocytopenia followed by rising renal indices, dynamic laboratory assessment may offer more reliable clues than a single snapshot (33). We do not advocate routine mNGS or hantavirus screening for every patient with CT-suspected pancreatitis; rather, hantavirus work-up should be reserved for patients presenting with the constellation of fever, severe thrombocytopenia, and renal impairment, especially within endemic zones (15). When clinical, biochemical, and radiological findings are mutually contradictory, metagenomic next-generation sequencing represents a powerful adjunct diagnostic modality for severe undifferentiated febrile multi-organ injury, as demonstrated here by the timely identification of Seoul orthohantavirus and the guidance it provided for appropriate management.

## Data Availability

The authors acknowledge that the data presented in this study must be deposited and made publicly available in an acceptable repository, prior to publication. Frontiers cannot accept a manuscript that does not adhere to our open data policies.

## References

[1] Jiang H Zheng X Wang L Du H Wang P Bai X. Hantavirus infection: a global zoonotic challenge. Virol Sin. (2017) 32:32–43. 10.1007/s12250-016-3899-x 28120221 PMC6598904

[2] Kilit TP Kilit C Erarslan S. A rare cause of acute pancreatitis: hantavirus infection. Acta Gastroenterol Belg. (2017) 80:59–61. 29364099

[3] Yu X Wang M Kong Q. Viral pancreatitis: research advances and mechanisms. Front Microbiol. (2023) 14:1326837. 10.3389/fmicb.2023.1326837 38420214 PMC10901110

[4] Puca E Harxhi A Pipero P Qyra E Stroni G Muco E. Pancreatitis in patients with hemorrhagic fever with renal syndrome: a five-year experience. J Infect Dev Ctries. (2017) 11:900–3. 10.3855/jidc.9567 31618190

[5] Tervo L Outinen T Kiekara T Tietäväinen J Paakkala A Pörsti I. The presence of intraperitoneal, retroperitoneal and pleural fluid in acute puumala hantavirus infection. Infect Dis (Lond). (2023) 55:207–15. 10.1080/23744235.2022.2160010 36562294

[6] Ye Z Liu X Ding S Lu L Zhang T Zhou W. Incidence rate of hemorrhagic fever with renal syndrome complicated with acute pancreatitis: a meta-analysis. Front Med (Lausanne). (2024) 11:1442276. 10.3389/fmed.2024.1442276 39502643 PMC11534719

[7] Zhong L Shi H Li H Xiao L Peng Q Liu Z. Acute pancreatitis and leukemoid reaction as the presenting manifestation of hemorrhagic fever with renal syndrome: a case report. Infect Drug Resist. (2024) 17:5347–54. 10.2147/IDR.S499197 39635289 PMC11616425

[8] Simner PJ Miller S Carroll KC. Understanding the promises and hurdles of metagenomic next-generation sequencing as a diagnostic tool for infectious diseases. Clin Infect Dis. (2018) 66:778–88. 10.1093/cid/cix881 29040428 PMC7108102

[9] Wang W-J Zhao J Yang J-S Liang M-M Ni M-Y Yang J-H. Clinical analysis of patients with acute pancreatitis complicated with hemorrhagic fever with renal syndrome and acute biliary pancreatitis. Medicine (Baltimore). (2020) 99:e18916. 10.1097/MD.0000000000018916 32000399 PMC7004732

[10] Zhang S Shi L Jiang X Yang X Wu L Wang C. Hyperamylasemia caused by the hantaan virus: a retrospective study of 101 patients with hemorrhagic fever with renal syndrome in west China. BioMed Res Int. (2022) 2022:4942697. 10.1155/2022/4942697 PMC9250431 35789642

[11] Bui-Mansfield LT Cressler DK. Imaging of hemorrhagic fever with renal syndrome: a potential bioterrorism agent of military significance. Mil Med. (2011) 176:1327–34. 10.7205/MILMED-D-11-00048 22165665

[12] Markotić A. Immunopathogenesis of hemorrhagic fever with renal syndrome and hantavirus pulmonary syndrome. Acta Med Croat. (2003) 57:407–14. 15011469

[13] Khaiboullina SF Levis S Morzunov SP Martynova EV Anokhin VA Gusev OA. Serum cytokine profiles differentiating hemorrhagic fever with renal syndrome and hantavirus pulmonary syndrome. Front Immunol. (2017) 8:567. 10.3389/fimmu.2017.00567 28572804 PMC5435745

[14] Tai P-W Chen L-C Huang C-H. Hanta hemorrhagic fever with renal syndrome: a case report and review. **J** Microbiol Immunol Infect. (2005) 38:221–3. 15986074

[15] Park ME Kim DY Seo J-W Yun NR Lee YM Kim CM. Analysis of clinical and laboratory profiles of patients hospitalized with hemorrhagic fever with renal syndrome in southwestern South Korea. Am J Trop Med Hyg. (2025) 112:161–6. 10.4269/ajtmh.24-0019 39481131 PMC11720790

[16] Chen T-C Perng D-S Tsai J-J Lu P-L Chen T-P. Dengue hemorrhagic fever complicated with acute pancreatitis and seizure. J Formos Med Assoc. (2004) 103:865–8. 15549156

[17] Alnuaimi MK Alsubai JA Alnuaimi A. Pancreatitis as a rare complication of dengue fever: a case report and review of the literature. Cureus. (2025) 17:e82725. 10.7759/cureus.82725 40400801 PMC12094532

[18] Jakab F Sebok J Szántó Z Hang D Imre M Németh V. Dobrava-Belgrade hantavirus infection mimics acute appendicitis. J Clin Virol. (2011) 50:164–6. 10.1016/j.jcv.2010.10.009 21075678

[19] Latus J Fritzenkötter M Schmidt-Chanasit J Tenner-Racz K Leibold T Kimmel M. Hantavirus and acute appendicitis—the diagnosis behind the diagnosis? J Clin Virol. (2012) 53:156–8. 10.1016/j.jcv.2011.11.003 22169597

[20] Zhu Y Chen Y-X Zhu Y Liu P Zeng H Lu N-H. A retrospective study of acute pancreatitis in patients with hemorrhagic fever with renal syndrome. BMC Gastroenterol. (2013) 13:171. 10.1186/1471-230X-13-171 24345089 PMC3878513

[21] Tian B Qu D Sasaki A Chen J Deng B. Acute pancreatitis in patients with severe fever with thrombocytopenia syndrome virus infection. Pancreatology. (2020) 20:1631–6. 10.1016/j.pan.2020.09.024 33092955

[22] Parenti DM Steinberg W Kang P. Infectious causes of acute pancreatitis. Pancreas. (1996) 13:356–71. 10.1097/00006676-199611000-00005 8899796

[23] Bargo EG Manubens TL Hernández JAH Gris PP. Acute pancreatitis caused by hepatitis B virus. Medicina Clínica. (1993) 101:799. 8114548

[24] Jain P Nijhawan S Rai RR Nepalia S Mathur A. Acute pancreatitis in acute viral hepatitis. World J Gastroenterol. (2007) 13:5741–4. 10.3748/wjg.v13.i43.5741 17963301 PMC4171261

[25] Sudhamshu KC Khadka S Sharma D Chataut SP. Acute pancreatitis in acute viral hepatitis. JNMA J Nepal Med Assoc. (2011) 51:7–10. 10.31729/jnma.20 22335088

[26] Wang C Fu B Su D Huang P Fu X. Acute pancreatitis and recurrent acute pancreatitis in children: a 10-year retrospective study. Gastroenterol Res Pract. (2022) 5505484:1–6. 10.1155/2022/5505484 PMC9337950 35911080

[27] Tervo L Outinen TK Mäkelä S Mustalahti J Huhtala H Pörsti I. Alcohol consumption and its influence on the clinical picture of puumala hantavirus infection. Viruses. (2022) 14:500. 10.3390/v14030500 35336910 PMC8948946

[28] Zhang D Yang QW. Construction and quality assurance of metagenomic next-generation sequencing platform in pathogen detection. Zhonghua Yi Xue Za Zhi. (2023) 103:1092–7. 10.3760/cma.j.cn112137-20220825-01801 37055227

[29] Hardick J Anantharam R Lu J Salzberg SL Rothman RE Fenstermacher KZJ. Comparison of unbiased metagenomic next generation sequencing to targeted multiplex diagnostic assays for the detection of respiratory viruses. PLoS One. (2026) 21:e0347750. 10.1371/journal.pone.0347750 42096470 PMC13152136

[30] Li Y Bian W Wu S Zhang J Li D. Metagenomic next-generation sequencing for mycobacterium tuberculosis complex detection: a meta-analysis. Front Public Health. (2023) 11:1224993. 10.3389/fpubh.2023.1224993 37637815 PMC10450767

[31] Peng J-M Du B Qin H-Y Wang Q Shi Y. Metagenomic next-generation sequencing for the diagnosis of suspected pneumonia in immunocompromised patients. J Infect. (2021) 82:22–7. 10.1016/j.jinf.2021.01.029 33609588

[32] Liu H Xu T Fu H Dai B Xie Y. Application of metagenomic next-generation sequencing in HIV-infected patients with bloodstream infections. Infect Drug Resist. (2025) 18:2389–99. 10.2147/IDR.S509665 40357419 PMC12068312

[33] Shi H Du F Wang T Gu Z Ruan T Zhao Q Xu R Wang Y Luo L Wang S. Laboratory data analysis of hemorrhagic fever with renal syndrome patients to predict disease severity and patient prognosis. J Clin Lab Anal (2025) 39:e70126. 10.1002/jcla.70126 41144944 PMC12699194

