# Supplementary material for: Case Report: Hemorrhagic fever with renal syndrome caused by Seoul orthohantavirus mimicking severe acute pancreatitis: dynamic imaging evolution and pathogen confirmation by mNGS
Source: Front Med (Lausanne). 2026 Oct 28;13:1995402. doi: 10.3389/fmed.2026.1995402 (PMC13751596; doi:10.3389/fmed.2026.1995402)
Supplement: Supplementary file 1 [file Supplementaryfile1.docx]

**CARE Checklist of information to include when writing a case report**

| **Topic** | **Item** | **Checklist item description** | **Reported in manuscript section** |
| --- | --- | --- | --- |
| Title | 1 | The diagnosis or intervention of primary focus followed by the words “case report” | Title page |
| Key Words | 2 | 2 to 5 key words that identify diagnoses or interventions in this case report, including "case report" | Keywords section |
| Abstract(no references) | 3a | Introduction: What is unique about this case and what does it add to the scientific literature? | Abstract section |
|  | 3b | Main symptoms and/or important clinical findings | Abstract section |
|  | 3c | The main diagnoses, therapeutic interventions, and outcomes | Abstract section |
|  | 3d | Conclusion—What is the main “take‑away” lesson(s) from this case? | Abstract section |
| Introduction | 4 | One or two paragraphs summarizing why this case is unique (may include references) | Introduction section |
| Patient Information | 5a | De‑identified patient specific information | Case presentation |
|  | 5b | Primary concerns and symptoms of the patient | Case presentation |
|  | 5c | Medical, family, and psycho‑social history including relevant genetic information | Case presentation; no relevant family/psychosocial history mentioned in manuscript |
|  | 5d | Relevant past interventions with outcomes | Case presentation, Table 1 (Diagnostic timeline) |
| Clinical Findings | 6 | Describe significant physical examination (PE) and important clinical findings | Case presentation, Table 1 (Diagnostic timeline), Laboratory and imaging findings subsection |
| Timeline | 7 | Historical and current information from this episode of care organized as a timeline | Case presentation, Table 1 (Diagnostic timeline) |
| Diagnostic Assessment | 8a | Diagnostic testing (such as PE, laboratory testing, imaging, surveys) | Laboratory and imaging findings subsection, Table1, Table2, mNGS, CT and ultrasound imaging |
|  | 8b | Diagnostic challenges (such as access to testing, financial, or cultural) | Discussion section (diagnostic anchoring bias, radiological mimicry of severe acute pancreatitis) |
|  | 8c | Diagnosis (including other diagnoses considered) | Case presentation; differential diagnosis: severe acute pancreatitis vs HFRS caused by Seoul orthohantavirus |
|  | 8d | Prognosis (such as staging in oncology) where applicable | Not applicable for this infectious case; full clinical recovery described in Case presentation |
| Therapeutic Intervention | 9a | Types of therapeutic intervention (such as pharmacologic, surgical, preventive, self‑care) | Hospital course and management subsection |
|  | 9b | Administration of therapeutic intervention (such as dosage, strength, duration) | Case presentation, Table1 timeline; drug dosage not reported in manuscript |
|  | 9c | Changes in therapeutic intervention (with rationale) | Hospital course and management subsection; therapy adjusted after mNGS confirmation of HFRS |
| Follow‑up and Outcomes | 10a | Clinician and patient‑assessed outcomes (if available) | Case presentation, Table1; full clinical recovery assessed by clinicians; no patient‑reported outcome provided |
|  | 10b | Important follow‑up diagnostic and other test results | Laboratory and imaging findings subsection, Table2, Figure2 pre‑discharge ultrasound; planned outpatient HBV and liver‑function follow‑up |
|  | 10c | Intervention adherence and tolerability (How was this assessed?) | Not applicable; patient completed in‑hospital treatment and discharged in good condition |
|  | 10d | Adverse and unanticipated events | Not applicable; no adverse or unexpected therapy‑related events reported |
| Discussion | 11a | A scientific discussion of the strengths AND limitations associated with this case report | Discussion section (limitations paragraph) |
|  | 11b | Discussion of the relevant medical literature with references | Discussion section, throughout with citations |
|  | 11c | The scientific rationale for any conclusions (including assessment of possible causes) | Discussion section; differentiation of HFRS‑mediated pancreatitis‑like imaging; evaluation of HBV co‑infection |
|  | 11d | The primary “take‑away” lessons of this case report (without references) in a one paragraph conclusion | Final concluding paragraph of Discussion |
| Patient Perspective | 12 | The patient should share their perspective in one to two paragraphs on the treatment(s) they received | Not applicable; patient perspective was not collected for this report |
| Informed Consent | 13 | Did the patient give informed consent? Please provide if requested | Ethics statement section |
